# The mechanism of geniposide in patients with COVID-19 and atherosclerosis: A pharmacological and bioinformatics analysis

**DOI:** 10.1097/MD.0000000000039065

**Published:** 2024-08-02

**Authors:** Lijin Qing, Wei Wu

**Affiliations:** a First Affiliated Hospital of Guangzhou University of Chinese Medicine, Guangzhou, Guangdong 510405, China.

**Keywords:** atherosclerosis, bioinformatics, COVID-19, geniposide, system pharmacology

## Abstract

In patients with severe acute respiratory syndrome coronavirus 2 (which causes coronavirus disease 2019 [COVID-19]), oxidative stress (OS) is associated with disease severity and death. OS is also involved in the pathogenesis of atherosclerosis (AS). Previous studies have shown that geniposide has anti-inflammatory and anti-viral properties, and can protect cells against OS. However, the potential target(s) of geniposide in patients with COVID-19 and AS, as well as the mechanism it uses, are unclear. We combined pharmacology and bioinformatics analysis to obtain geniposide against COVID-19/AS targets, and build protein–protein interaction network to filter hub genes. The hub genes were performed an enrichment analysis by ClueGO, including Gene Ontology and KEGG. The Enrichr database and the target microRNAs (miRNAs) of hub genes were predicted through the MiRTarBase via Enrichr. The common miRNAs were used to construct the miRNAs-mRNAs regulated network, and the miRNAs’ function was evaluated by mirPath v3.0 software. Two hundred forty-seven targets of geniposide were identified in patients with COVID-19/AS comorbidity by observing the overlap between the genes modulated by geniposide, COVID-19, and AS. A protein–protein interaction network of geniposide in patients with COVID-19/AS was constructed, and 27 hub genes were identified. The results of enrichment analysis suggested that geniposide may be involved in regulating the OS via the FoxO signaling pathway. MiRNA-mRNA network revealed that hsa-miR-34a-5p may play an important role in the therapeutic mechanism of geniposide in COVID-19/AS patients. Our study found that geniposide represents a promising therapy for patients with COVID-19 and AS comorbidity. Furthermore, the target genes and miRNAs that we identified may aid the development of new treatment strategies against COVID-19/AS.

## 1. Introduction

Coronaviruses (CoVs) are members of the subfamily *Coronavirinae* in the family *Coronaviridae*.^[1]^ CoVs are single-stranded RNA viruses, which can infect multiple systems in animals and humans, including the respiratory, gastrointestinal, hepatic, and central nervous systems.^[2]^ At least 3 pathogenic human CoVs have caused major epidemics in recent history; the severe acute respiratory syndrome coronavirus (SARS-CoV) in 2002; the Middle East respiratory syndrome coronavirus in 2012^[3]^, and the severe acute respiratory syndrome coronavirus 2 (SARS-CoV-2) in 2019. SARS-CoV-2 is responsible for coronavirus disease 2019 (COVID-19). The clinical features of COVID-19 are varied and nonspecific. Disease presentation can range from asymptomatic to severe pneumonia and death,^[4]^ and new mutant strains are still being routinely identified. Studies have reported that individuals most severely affected by COVID-19 are over 60 years of age.^[5,6]^ Patients with a history of underlying health conditions such as cardiovascular disease, hypertension, chronic obstructive pulmonary disease, and diabetes are also more likely to experience COVID-19 complications, which manifest as respiratory failure, septic shock, and multiple organ failure.^[7]^

Excess mortality from cardiovascular disease during influenza epidemics was initially recognized in the 20th century because of the nature of the virus, and reports indicated that COVID-19 has a similar tendency.^[8]^ The typical symptoms of COVID-19 range from asymptomatic infections to mild respiratory symptoms to severe pneumonia. The long-term effect of COVID-19 has been proved to have negative effects on human health, involving multiple systems, including cardiovascular.^[9]^ For people with cardiovascular disease or compromised immunity, active prevention remains important after the COVID-19 initial infection. Severe COVID-19 symptoms are caused by an intense inflammatory response and cytokine overproduction.^[10]^ Atherosclerosis (AS) is a common pathogenesis of cardiovascular diseases and AS is characterized by the formation of lesions in the wall of the artery and persistent chronic inflammation.^[11]^ The development of AS involves complex pathophysiological pathways and pro/anti-inflammatory cytokines.^[12]^ In patients with COVID-19, the high incidence of cardiovascular symptoms is also due to the systemic inflammatory response and immune system disorders.^[13]^ After infecting respiratory epithelial cells, SARS-CoV-2 activates T helper 1 cells to secrete proinflammatory cytokines.^[14]^ Proinflammatory cytokines such as interferon-γ, interleukin (IL)-1β, IL-6, and tumor necrosis factor (TNF)-α induce excessive macrophage activation and trigger the apoptosis of epithelial cells,^[15,16]^ which is also a sign of AS progression. Therefore, anti-inflammatory medications may help treat AS patients with SARS-CoV-2 infection.

Geniposide is an iridoid glycoside compound, which is extracted from the dried, mature fruit of *Gardenia jasminoides* (common name gardenia or cape jasmine). Geniposide has been shown to exert anti-inflammatory and antiogxidant effects in various diseases, including cardiovascular disease, diabetes, nonalcoholic fatty liver disease, Alzheimer disease and Parkinson disease.^[11,17–19]^ A previous study of AS demonstrated that geniposide, combined with notoginsenoside R1, can effectively inhibit AS-associated inflammation and apoptosis by activating the AMPK/mTOR/Nrf2 signaling pathway. This in turn results in the inhibition of the NOD-like receptor family pyrin domain-containing 3 inflammasome and the Bax/Bcl2/caspase-3 pathway which is the key point in the inflammation.^[20]^ Geniposide has also been shown to alleviate AS injury caused by inflammation by regulating the miR-101/MKP-1/p38 pathway.^[21]^ In addition, researchers provide evidence that geniposide can improve the immune response by regulating humoral immunity and cellular immunity, and enhance pathogen resistance.^[22]^ Another study revealed that the geniposide regulates calcium signaling pathway essential for influenza A virus replication.^[23]^ Because of these anti-inflammatory and anti-virus effects, geniposide is likely to have considerable therapeutic potential. However, the underlying mechanism remains unclear.

**Figure 1.**
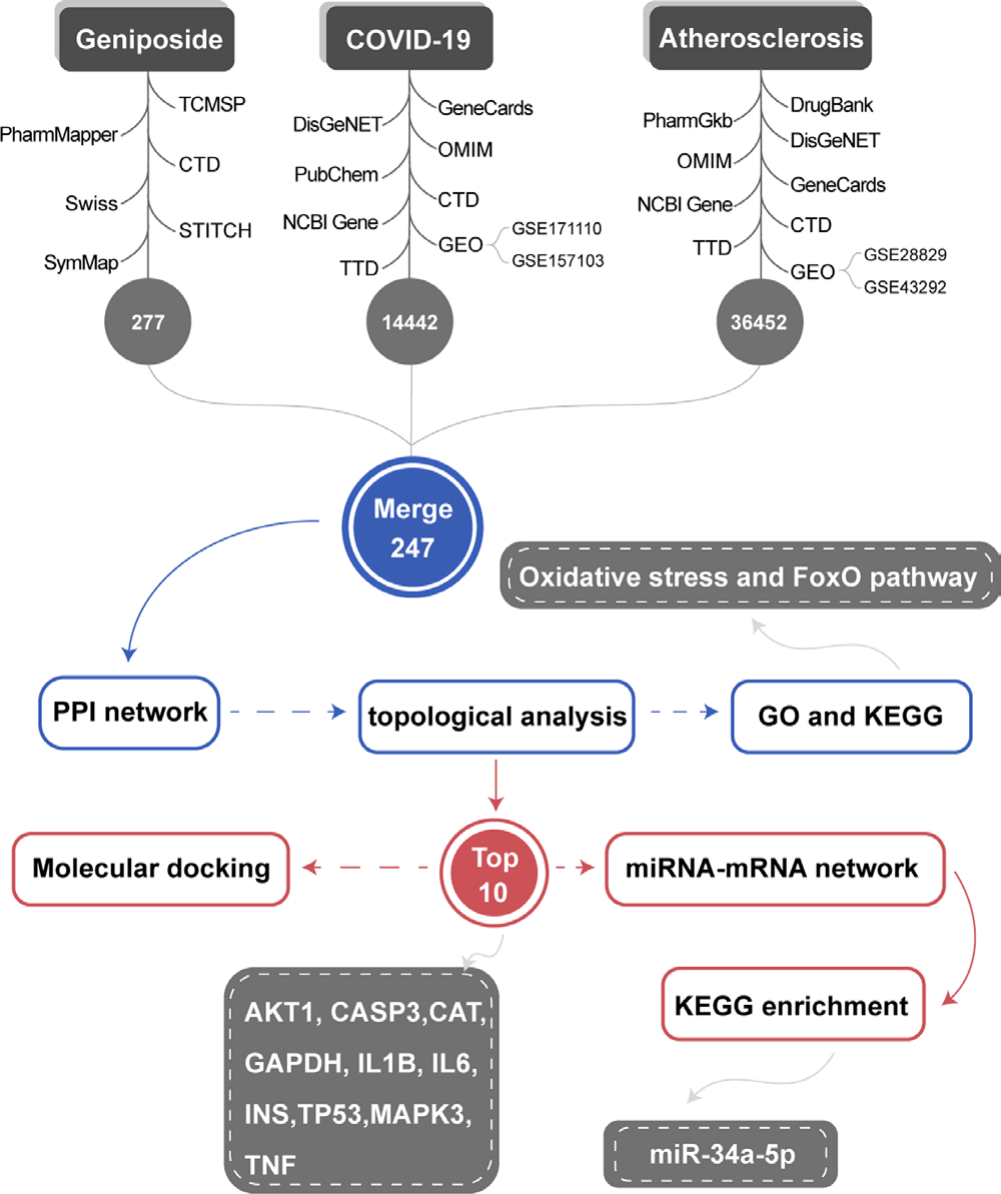
Study workflow. COVID-19 = coronavirus disease 2019, GO = gene ontology, KEGG = Kyoto Encyclopedia of Genes and Genomes, PPI = protein–protein interaction.

To explore the potential biomarkers of geniposide in patients with COVID-19 and AS comorbidity, we screened the possible pharmacological mechanism of geniposide in AS patients with COVID-19 using a combination of system pharmacology and bioinformatics approaches. We extracted common genes and constructed a protein–protein interaction (PPI) network of geniposide in patients with AS and COVID-19. In addition, common miRNAs were used to construct the miRNA-mRNA regulatory network. The results we concluded may aid the development of new treatment strategies against COVID-19/AS. The workflow of the study is shown in Figure 1.

## 2. Materials and methods

### 2.1. Predicting the function of geniposide targets

The 2 dimentional (2D) structure of geniposide was obtained from the PubChem compound database (https://pubchem.ncbi.nlm.nih.gov/). Different types of pharmacological targets related to geniposide were collected from the following databases: Traditional Chinese Medicine Systems Pharmacology Database and Analysis Platform (TCMSP, http://tcmspw.com/),^[24]^ PharmMapper (http://www.lilabecust.cn/pharmmapper/),^[25]^ Swiss Target Prediction (http://www.swisstargetprediction.ch/),^[26]^ Symptom Mapping (SymMap, https://www.Symmap.org/),^[27]^ Chemical Association Networks (STITCH, http://stitch.embl.de/),^[28]^ and Comparative Toxicogenomics Database (CTD, http://ctdbase.org/).^[29]^ Target protein names were converted into the corresponding gene symbols using the UniProt database (https://www.uniprot.org/), limiting data to “Homo sapiens.”

### 2.2. Screening of COVID-19– or AS-related targets

The COVID-19–related microarray datasets, GSE157103^[30]^ and GSE171110,^[31]^ were downloaded from the Gene Expression Omnibus (GEO, https://www.ncbi.nlm.nih.gov/geo/). GSE157103 contained data on 100 COVID-19-positive patients and 26 healthy individuals; GSE171110 contained data on 44 COVID-19–positive patients and 10 healthy individuals. We converted the probes into gene symbols and excluded probes containing multiple genes. Furthermore, the batch effect was removed using the “sva” package in R software. The “limma” package in R software was applied to identify COVID-19–related differentially expressed genes (DEGs), which were screened by adjusted *P* value <.05 and |log2FC| >1. In addition, COVID-19–related targets were obtained from 7 open-source databases: OMIM (https://omim.org/), NCBI Gene (https://www.ncbi.nlm.nih.gov/), TTD (http://db.idrblab.net/), PubChem, CTD (http://ctdbase.org/), DisGeNET (http://www.disgenet.org), and GeneCards (https://www.genecards.org/).

The AS-related microarray datasets, GSE28829^[32]^ and GSE43292,^[33]^ were also downloaded from the GEO. GSE28829 contained data from 13 patients with early-stage carotid AS and 16 patients with advanced-stage carotid AS; GSE43292 contained data on 32 patients with early-stage and 32 patients with advanced-stage carotid AS. AS-related DEGs were acquired after processing AS-related data, as in the case of analyzing COVID-19-related data. AS-related targets were also obtained from 8 open-source databases: CTD, DisGeNET, DrugBank (https://go.drugbank.com/), GeneCards, OMIM, TTD, PharmGKB (https://www.pharmgkb.org/), and NCBI Gene.

### 2.3. Construction of the PPI network

A Venn diagram (http://bioinformatics.psb.ugent.be/Webtools/Venn/) was used to identify any overlap between genes induced by geniposide, COVID-19, and AS. Next, the species of interest was defined as “Homo sapiens” and the STRING database (https://string-db.org/) was used to construct the PPI network of geniposide in the treatment of patients with COVID-19/AS. The data file was downloaded in TSV format. The PPI network was analyzed and visualized using Cytoscape software; the nodes represent target proteins and the lines indicate interactions between them. Subsequently, topological analysis was performed using the CytoNCA plug-in in Cytoscape. Key parameters such as betweenness centrality (BC), closeness centrality (CC), degree centrality (DC), eigenvector centrality (EC), local average connectivity-based method (LAC), and network centrality (NC) were used to filter out target genes for which each parameter value was greater than the median; overlapping genes were selected to construct a sub-network. Using the same steps, the sub-network was screened a second time to select hub gene for further study.

### 2.4. Enrichment analysis of hub genes

Gene Ontology (GO) and Kyoto Encyclopedia of Genes and Genomes (KEGG) pathway enrichment analyses of hub genes were performed using the ClueGO plug-in in Cytoscape and visualized as a functionally grouped network.^[34]^ To explore the potential roles of these hub genes, which were shared between geniposide, COVID-19, and AS, a biological analysis was performed using ClueGO; a *P* value <.05 was considered as a measure of statistical significance.

### 2.5. Molecular docking

The 2D structure of geniposide was retrieved from the PubChem database (https://pubchem.ncbi.nlm.nih.gov/) and downloaded in a SDF format file. Chem3D Ultra was used to convert the 2D geniposide structure into a 3D structure; the data file was saved in MOL format as a docking ligand. Hub genes with the most significant centrality in the PPI network were selected as docking receptors, and the corresponding target protein structures were downloaded from the PDB database (https://www.rcsb.org/). Next, water molecules and original ligands were removed using PYMOL, and hydrogen atoms were added using AutoDockTools software. The docking ligand was also imported to set the spatial docking grid between the ligand and receptor. Eventually an output ligand file was generated in PDBQT format. AutoDock Vina was used to perform molecular docking and calculate the free binding energy and root mean square deviation. The PYMOL visualization system was employed to predict the optimal docking conformation.

### 2.6. Identification of shared miRNAs and miRNAs-mRNA network construction

Enrichr is a comprehensive tool for gene enrichment analysis, which contains a large number of gene libraries and annotated gene sets such as MiRTarBase. MiRTarBase is a curated experimentally supported database of human miRNAs and their disease associations.^[35]^ After setting the *P* value <.05 as a screening condition, hub genes of geniposide in the treatment of COVID-19/AS and the related miRNAs were obtained. Only the miRNAs associated with COVID-19 and AS were further analyzed. Shared miRNAs were used to construct the mRNA-miRNA regulatory network, which was visualized using Cytoscape software. To explore the function of the selected miRNAs, DIANA-mirPath software was used to perform enrichment analysis.

## 3. Results

### 3.1. Identification of target genes associated with geniposide treatment and COVID-19/AS

**Figure 2.**
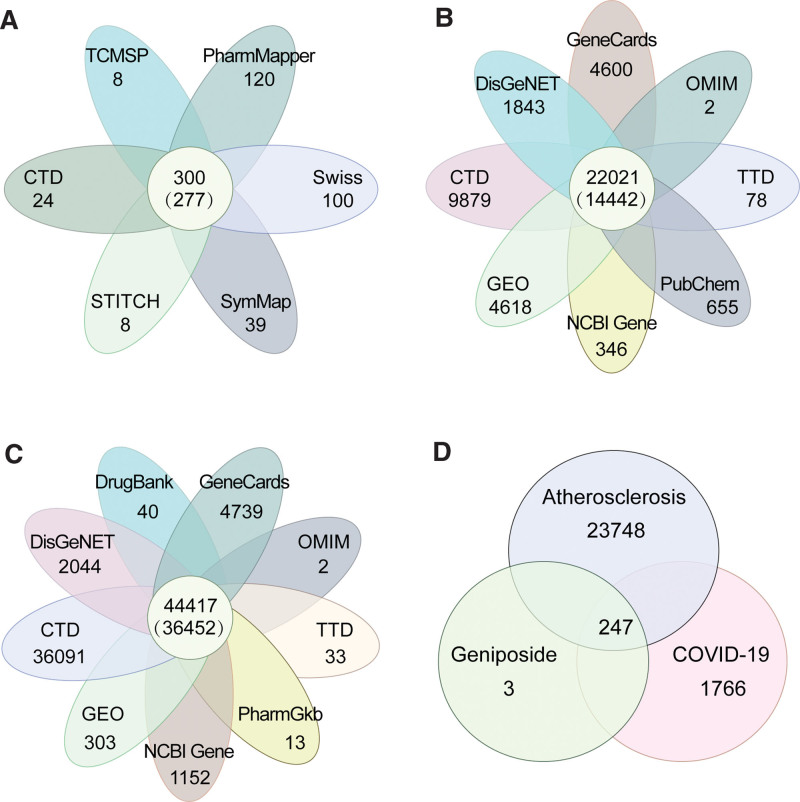
(A) The target genes associated with geniposide application in humans were obtained from 6 databases. (B) The target genes related to COVID-19 were selected from 2 GEO datasets and 7 databases. (C) AS-related target genes were obtained from 2 GEO datasets and 8 databases. (D) A Venn diagram depicting shared target genes between the geniposide, COVID-19, and AS conditions. AS = atherosclerosis, COVID-19 = coronavirus disease 2019, CTD = Comparative Toxicogenomics Database, GEO = Gene Expression Omnibus, TCMSP = Traditional Chinese Medicine Systems Pharmacology Database and Analysis Platform.

Six databases were used to obtain expression data on target genes related to geniposide treatment: TCMSP (8), PharmMapper (120), Swiss Target Prediction (100), SymMap (39), STITCH (9), and CTD (24). The genes potentially targeted by geniposide treatment were identified by eliminating duplicate and nonstandard genes, which resulted in the identification of 278 target genes (Fig. 2A).

**Figure 3.**
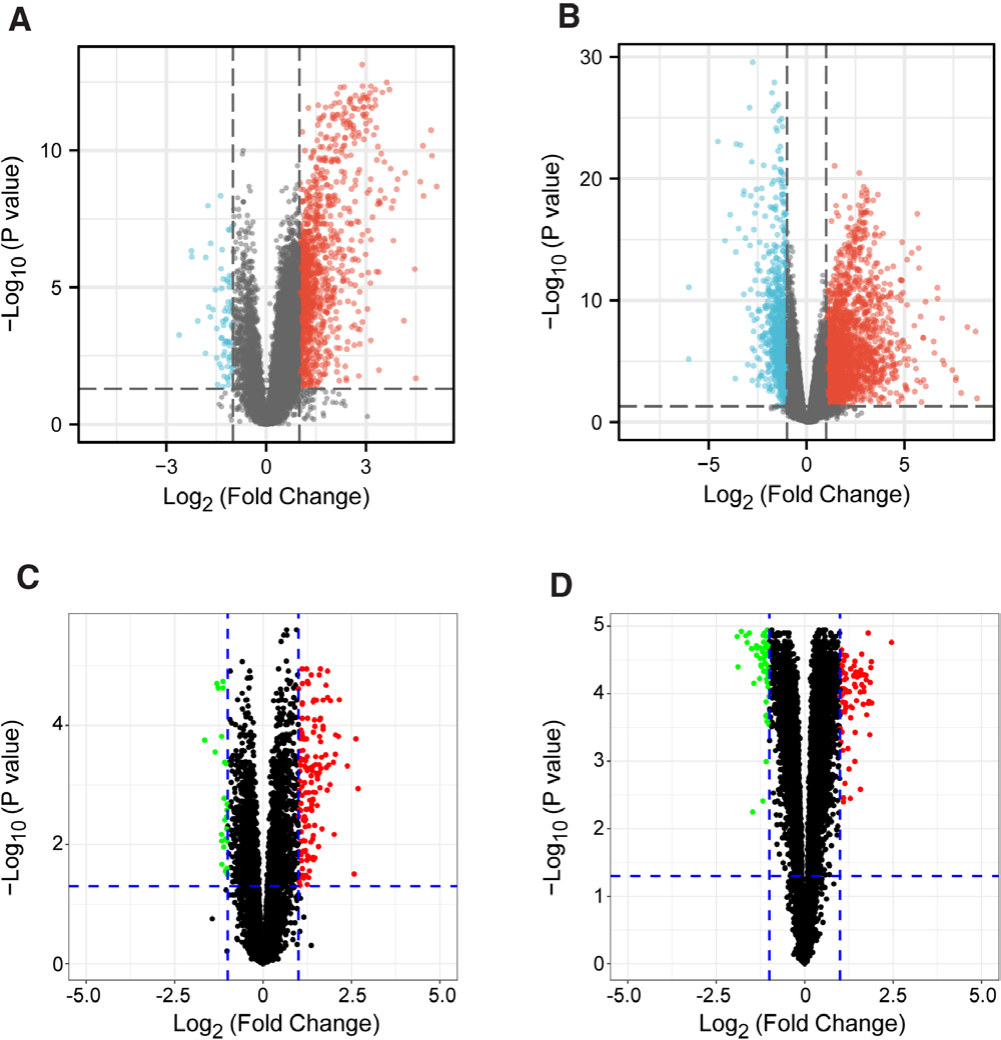
Volcano plots of DEGs between patients with AS or COVID-19 (*P* value < .05 and |log2FC| >1). Red and blue dots represent the up-regulated and down-regulated genes, respectively, while the black dots represent genes that exhibited no significant difference in expression between patients with AS and those with COVID-19. (A) The GSE157103 dataset. (B) The GSE171110 dataset. (C) The GSE28829 dataset. (D) The GSE43292 dataset. AS = atherosclerosis, COVID-19 = coronavirus disease 2019, DEGs = differentially expressed genes.

DEGs between patients with COVID-19 and healthy individuals were obtained by analyzing the GSE157103 and GSE171110 datasets, which yielded 1316 and 3302 target genes, respectively. Figure 3A, B shows a comparison of gene expression between patients with COVID-19 and healthy individuals; red indicates up-regulated genes, green indicates down-regulated genes, and black indicates no difference in gene expression.

COVID-19–related genes were also obtained from 7 open-source databases: OMIM (2), NCBI Gene (346), TTD (78), PubChem (655), CTD (9879), DisGeNET (1843), GeneCards (4600). Potential targets were identified by eliminating duplicate and nonstandard targets, which resulted in the identification of 14,442 targets (Fig. 2B).

Data on DEGs between patients with early-stage and late-stage carotid AS were obtained from the GSE28829 (171 targets) and GSE43292 (132 targets), respectively; the volcano plot of these data is shown in Figure 3C, D.

AS-related genes were also selected from 8 open-source databases: CTD (36091), DisGeNET (2044), DrugBank (40), GeneCards (4739), OMIM (2), TTD (33), PharmGKB (13), and NCBI Gene (1152). Potential targets were identified by eliminating duplicate and nonstandard targets, which resulted in the identification of 36,452 target genes (Fig. 2C).

### 3.2. PPI network construction

**Figure 4.**
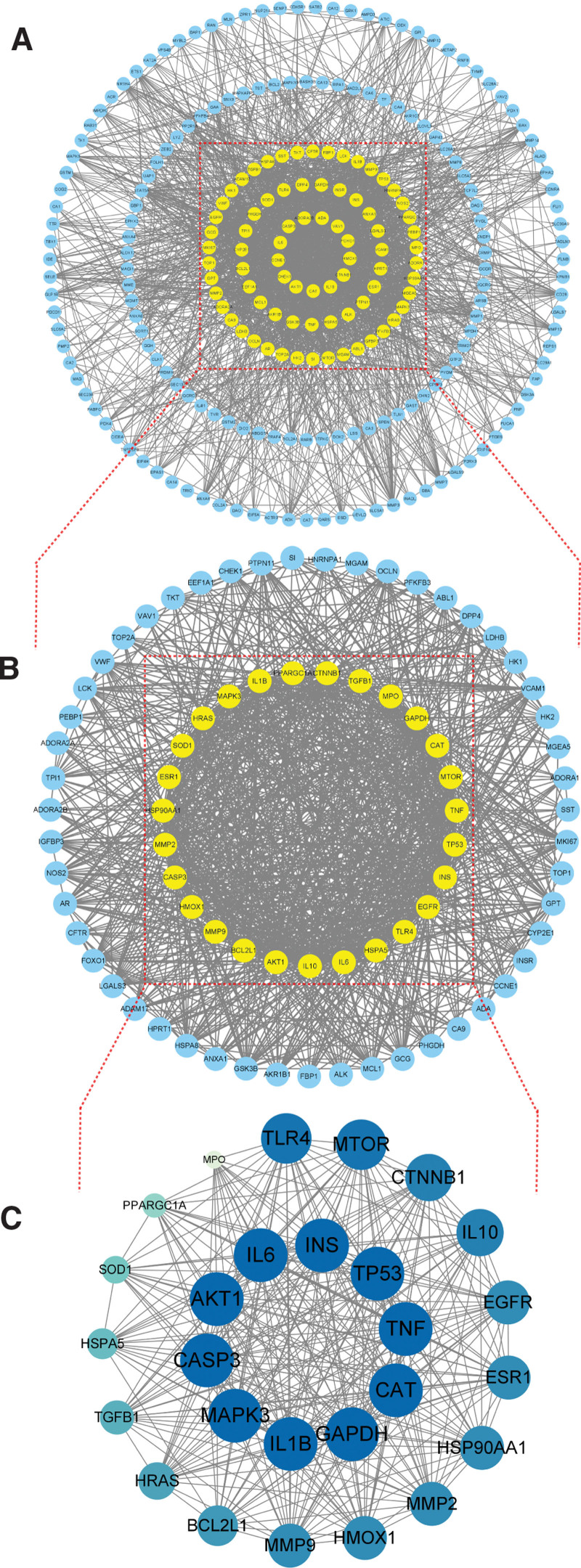
PPI network construction and hub genes of geniposide against COVID-19/AS. The thickness of the line is positively correlated with the intensity of the interaction between the nodes (proteins). Node size is proportional to the significance of a particular protein in a given physiological context. (A) PPI network of geniposide in relation to COVID-19/AS. (B) A PPI subnetwork of significant proteins extracted from the main PPI network in (A). (C) A further PPI subnetwork of 27 hub genes potentially modulated by geniposide in patients with COVID-19/AS, extracted from the PPI subnetwork shown in (B). AS = atherosclerosis, COVID-19 = coronavirus disease 2019, PPI = protein–protein interaction.

Two hundred forty-seven shared target genes (Supplementary Table S1, Supplemental Digital Content, http://links.lww.com/MD/N279) between the geniposide, COVID-19, and AS conditions, were considered as the genes that geniposide could modulate to potentially treat patients with COVID-19 and AS (Fig. 2D). We then imported the 247 target genes into STRING to construct a PPI network diagram (Fig. 4A). The PPI network had a total of 239 nodes and 2225 edges. We then analyzed the topological characteristics of the network graph and selected the index above the median values of BC, CC, DC, EC, LAC, NC as the key targets. The first screening was based on the thresholds of BC ≥82.124, CC ≧0.427, DC ≧12.000, EC ≥0.025, LAC ≥5.455, and NC ≥6.625; a total of 78 nodes and 1066 edges were obtained (Fig. 4B). Then, using BC ≧12.276, CC ≧0.588, DC ≧23.000, EC ≥0.096, LAC ≥16.234, and NC ≥19.024 as the screening threshold, a total of 27 nodes and 608 edges were scored; the larger and more color-saturated the node, the more important its potential therapeutic role is in COVID-19/AS (Fig. 4C). Cytoscape software was used to visualize the degree values of the targets and to select the top genes modulated by geniposide that could be involved in the resolution of COVID-19/AS symptoms. The top 10 targets with the highest degree included *AKT1, CASP3, CAT, GAPDH, IL1B, IL6, INS, MAPK3, TNF,* and *TP53*, which as the crucial targets for geniposide in COVID-19/AS were selected for molecular docking and mRNA-miRNA network construction.

### 3.3. GO and KEGG enrichment analyses

**Figure 5.**
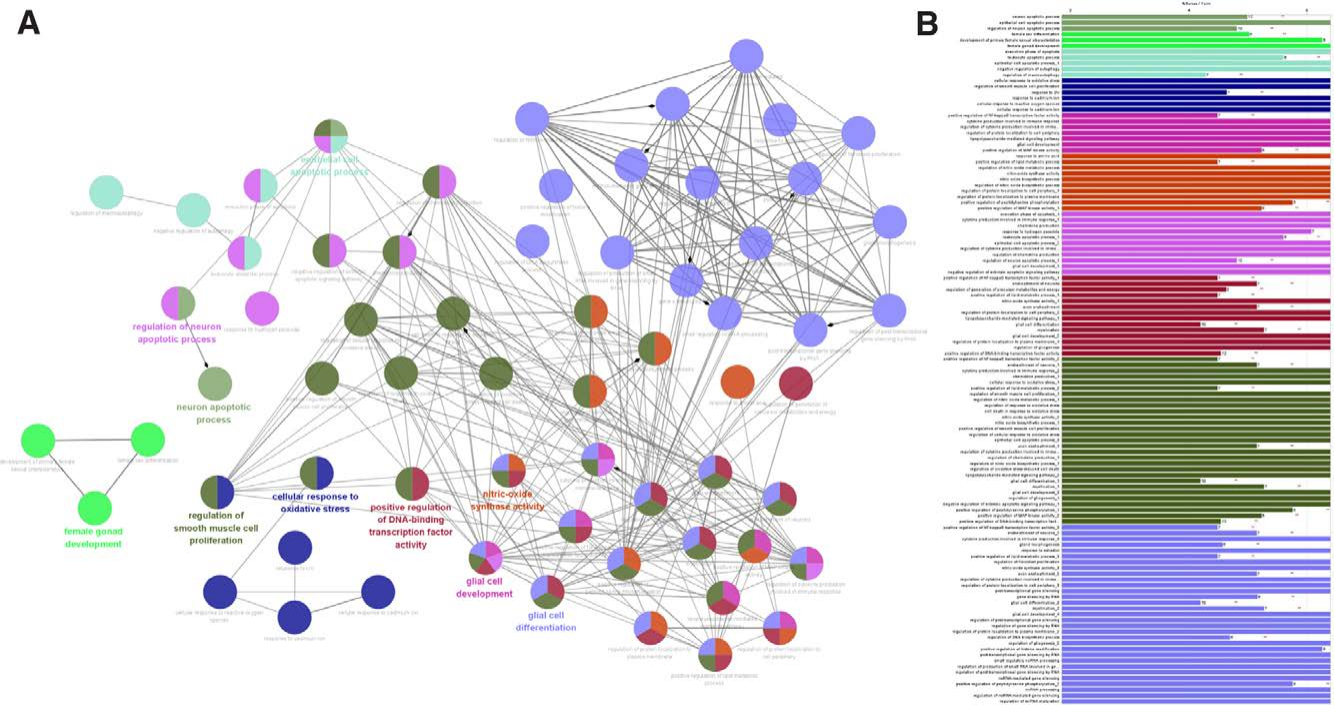
The interaction network of GO terms generated by ClueGO. (A and B) Results of GO enrichment analyses. The most significant term in each group is highlighted. **P < .05. GO = gene ontology.

**Figure 6.**
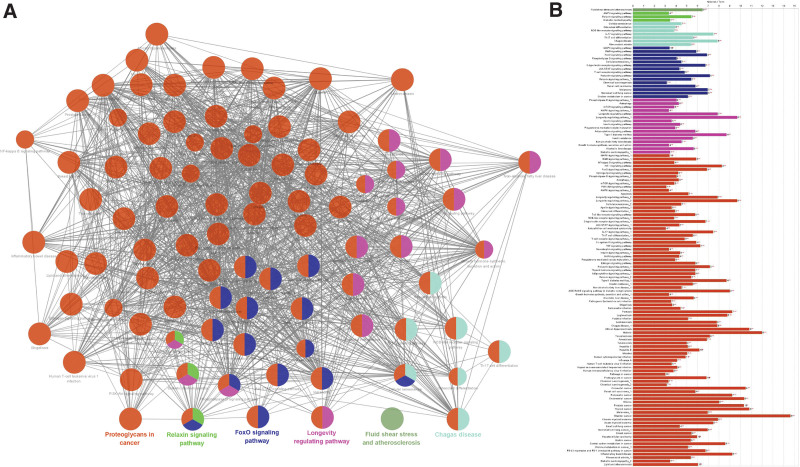
The interaction network of KEGG terms generated by ClueGO. (A and B) Results of KEGG enrichment analyses. The most significant term in each group is highlighted. **P < .05. KEGG = Kyoto Encyclopedia of Genes and Genomes.

To explore the pharmacological mechanism of geniposide in the treatment of COVID-19/AS, we performed GO and KEGG enrichment analyses using Cluego. GO analysis showed that the hub genes mainly participated in cellular response to oxidative stress, nitric-oxide synthase activity, and the positive regulation of DNA-binding transcription factor activity (Fig. 5). KEGG analysis showed that the hub genes mainly participated in the FoxO, longevity regulating, and relaxin signaling pathways (Fig. 6). The results of the enrichment analyses suggested that geniposide may exert therapeutic effects in patients with COVID-19/AS by mediating FoxO and other signaling pathways to regulate biological processes such as oxidative stress.

### 3.4. Molecular docking

**Figure 7.**
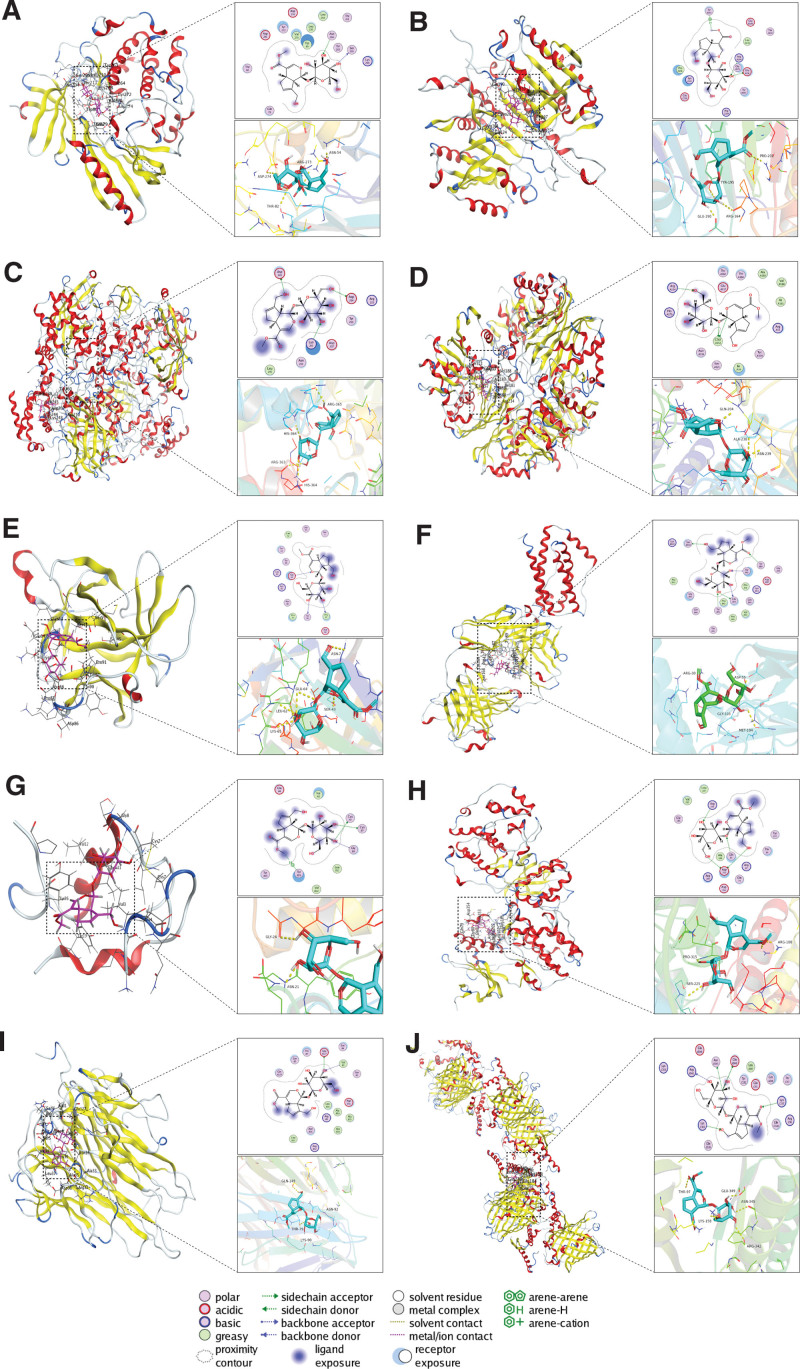
Molecular docking results. (A) Geniposide and the AKT1 protein. (B) Geniposide and the CASP3 protein. (C) Geniposide and the CAT protein. (D) Geniposide and the GAPDH protein. (E) Geniposide and the IL1B protein. (F) Geniposide and the IL6 protein. (G) Geniposide and the INS protein. (H) Geniposide and the MAPK3 protein. (I) Geniposide and the TNF protein. (J) Geniposide and the TP53 protein. GAPDH = glyceraldehyde-3-phosphate dehydrogenase.

The results of the molecular docking analysis showed that all top 10 targets had a good level of affinity for geniposide, with minimum binding energies <−5.0 kcal/mol and root mean square deviation <2 (Table 1). Moreover, the binding activities could be enhanced by linking to target protein residues in different ways, as shown in Figure 7. Based on the binding energy, geniposide had the strongest interaction with glyceraldehyde-3-phosphate dehydrogenase (GAPDH), but also had relatively good binding affinities for AKT1, CAT, TP53, CASP3, IL6, and MAPK3. Thus, geniposide may play a vital role in treating patients with COVID-19 and AS comorbidity by interacting with these targets. In terms of binding mode, geniposide was most closely bound to IL6 and TP53, while GAPDH, CAT, CASP3, MAPK3, IL1B, and INS were also favorable binding partners.

**Table 1:** Molecular docking of geniposide to its protein targets

Target protein	PDB ID	RMSD	Binding energy (kcal/mol)
AKT1	7NH5	1.32	−8.7
CASP3	7USP	1.75	−7.4
CAT	1DGH	0.00	−7.5
GAPDH	6YNH	1.70	−9.6
IL1B	9ILB	1.79	−6.4
IL6	4O9H	1.40	−7.3
INS	1A7F	1.33	−5.2
MAPK3	6GES	1.64	−7.3
TNF	1A8M	1.29	−6.0
TP53	7BWN	0.00	−7.5

GAPDH = glyceraldehyde-3-phosphate dehydrogenase, RMSD = root mean square deviation.

Six pairs of hydrogen bonds were formed between geniposide and the IL6 protein, which involved amino acid residues Gln 39, Gly 42, Ser 168, Tyr 95, and Tyr 89. Likewise, geniposide was connected to the TP53 protein by hydrogen bonding with amino acid residues Asn 345, Glu 349, Arg 342, Lys 107, and Lys 162. Geniposide hydrogen bonded to Asp 353, Gln 83, and Arg 165 of MAPK3. Geniposide formed a connection to Cys 7 and Gly 8 of the INS protein via hydrogen bonds and to Tyr 26 via π-H bonds. Csu 152 and Arg 234 of the GAPDH protein were also hydrogen bonded to geniposide. Geniposide hydrogen bonded with Asp 249, Asp 264, and Lys 243 of CAT, and Pro 87, Tyr 90, and Glu 64 of IL1B. The CASP3 protein not only formed hydrogen bonds with geniposide via Glu 124 and Arg 164 but also formed π-H bonds via Tyr 195. Geniposide was hydrogen bonded to Glu 127 and Val 1 of the TNF protein, and Ser 205 and Asn 204 of AKT1.

### 3.5. mRNA-miRNA network construction and miRNA enrichment analysis

**Figure 8.**
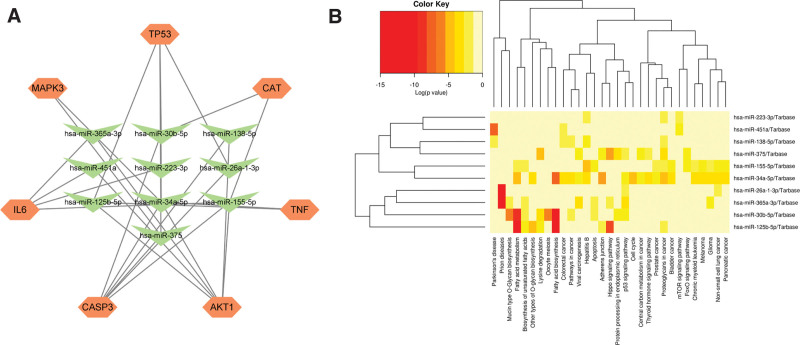
(A) Regulatory network of mRNA-miRNAs. (B) The functional enrichment analysis of 10 common miRNAs.

To identify changes occurring at the transcriptional level, we employed a network-based approach to explore the relationships between the top 10 genes and miRNAs, as shown in Figure 8A. It has been shown that one miRNA can regulate multiple genes, which indicates a strong level of interference between miRNAs. We performed KEGG pathway enrichment analysis of 10 common miRNAs using DIANA-mirPath software (Fig. 8B). We found that hsa-miR-34a-5p was most closely associated with the hub genes (Supplementary Table S2, Supplemental Digital Content, http://links.lww.com/MD/N280). Furthermore, the FoxO signaling pathway was identified by both the KEGG enrichment and common miRNA enrichment analyses.

## 4. Discussion

Because of population growth and aging, the absolute number of people living with cardiovascular disease people remain increase.^[36]^ The impact of the COVID-19 infection on longer-term trends in cardiovascular disease yet to be clearly established. On the other side, epidemiologic research showed that acute myocardial infarction is increased in COVID-19–infected heterozygous familial hypercholesterolemia patients, which is suggested that the risk for acute cardiovascular events is increased in the long-term.^[37]^

In this study, we identified 247 potential target genes modulated by geniposide in patients with COVID-19 and AS comorbidity and constructed a PPI network. We then analyzed the topological characteristics of the PPI network to visualize the degree value of targets and to select the top 10 genes, which included *AKT1, CASP3, CAT, GAPDH, IL1B, IL6, INS, MAPK3, TNF,* and *TP53*.

GO analysis revealed that geniposide treatment was associated with the cellular response to oxidative stress, nitric-oxide synthase activity, and positive regulation of DNA-binding transcription factor activity. The pathways highlighted by KEGG analysis were the FoxO, longevity regulating, and relaxin signaling pathways. Furthermore, the molecular docking analysis showed that geniposide may play a vital role in anti-COVID-19/AS comorbidity by interacting with 6 proteins, for which it exhibited good binding affinity. These results collectively suggest that geniposide has biological functionality, which could be harnessed to treat patients with COVID-19 and AS comorbidity.

### 4.1. Inflammation and oxidative stress play important roles in COVID-19 and AS comorbidity

Studies of COVID-19 pathophysiology have indicated that the increased expression of vascular and inflammatory factors (such as vascular cell adhesion molecule 1, IL-8, or monocyte-chemoattractant protein is accompanied by endothelial cell dysfunction and altered endothelial integrity.^[38,39]^ The presence of proinflammatory mediators drives a procoagulant state, which leads to excessive coagulation and eventually thrombosis.^[40]^ AS is a chronic inflammatory disease affecting the blood vessels, which leads to the formation of occlusive or rupture-prone lesions in the walls of large arteries.^[41]^ The relationship between lipoprotein particles, inflammation, and clotting cascades underlies the development of AS.^[14,42]^ In response to local inflammation and irritants, T cells, monocytes, macrophages, and platelets, as well as endothelial cells, vascular smooth muscle cells, and adipocytes, produce cytokines.^[43]^ The high level of proinflammatory cytokines promotes the development of AS.^[44]^ The cytokine-induced activation of endothelial cells disrupts normal endothelial function by changing the expression of adhesion molecules and chemokines, which speeds up plaque rupture. This ultimately causes blood clot formation, which is a characteristic of late-stage AS.^[45,46]^

The American Heart Association states that viral infection also reduces the cohesion of atherosclerotic plaques and encourages the progression of AS and coronary heart disease.^[47]^ For example, the inflammatory factors IL-1β and IL-6 play major roles in both AS and COVID-19.^[48]^ Similarly, IL-18 and IL-12, in combination with IL-1β, induce interferon-γ secretion and promote T helper cell and natural killer cell activity in both AS and COVID-19.^[49,50]^ In COVID-19 patients, the apoptosis of endothelial and epithelial cells in the lung tissues causes the release of chemokines, (mainly from the CXC family), which are also involved in atherosclerotic plaque formation, leading to monocyte/lymphocyte recruitment.^[51,52]^

Oxidative stress causes an increase in the generation of reactive oxygen species (ROS), which is accompanied by the reduced activity of innate antioxidant defense systems.^[53]^ ROS play an important role in the inflammatory response, apoptosis, cell growth, vascular tone, and the oxidation of low density lipoprotein cholesterol.^[54]^ ROS have also been shown to promote atherosclerotic plaque formation.^[55]^ There is growing evidence to support the association between oxidative stress and inflammation caused by high ROS production. Moreover, intracellular processes are highly influences by ROS concentrations.^[56]^ Oxidative stress is induced by high angiotensin (Ang) II and low Ang 1-7 levels, and negatively affects cell and tissue function.^[57]^ In addition, overactivation of the immune response, characterized by the production of proinflammatory cytokines, is common in patients with COVID-19 and is another trigger of endogenous oxidative stress.^[58]^ As mentioned earlier, inflammation and oxidative stress play important roles in COVID-19 and AS comorbidity. Therefore, reliable, selective biomarkers for these conditions are urgently needed.

### 4.2. Geniposide may modulate hub genes to participate in a variety of key processes

After combining the results of topological and molecular docking analyses, 10 hub genes were selected, including *AKT1, CASP3, CAT, GAPDH, IL1B, IL6, INS, MAPK3, TNF,* and *TP53*. As mentioned earlier, a significant increase in proinflammatory cytokines is implicated in the pathology of COVID-19 and AS.^[59,60]^ IL-1β, IL-6, and TNF-α are important inflammatory molecules, which induce abnormal proliferation of inflammatory cells by activating specific signaling pathways.^[61]^ Sul and Ra^[62]^ showed that modulating ROS, IL-1, IL-6, and TNF-α levels protected lung epithelial cells against oxidative stress and inflammation induced by lipopolysaccharide. In addition, suppression of lipopolysaccharide-induced macrophage inflammation and oxidative stress was accompanied by a decrease in proinflammatory factor levels.^[63]^ In a study of a traditional Chinese medicinal herb, used for the treatment of COVID-19 and its complications (such as AS and nephropathy), *MAPK3, TNF, GAPDH,* and *CASP3* were identified to have anti-inflammatory and antioxidant properties.^[64]^ We therefore have evidence to believe that there is an interaction between the inflammatory response and oxidative stress, which involves networks of reactions that serve to amplify each other. Geniposide has also been proven to exert anti-inflammatory effects by reducing the production of proinflammatory cytokines and the inhibiting p38MAPK/NF-κB signaling. Moreover, IL-6, IL-1β, AKT1, TNF-α, and MAPK3 may be the potential targets of geniposide.^[65]^ However, the mechanisms underlying the effects of geniposide on inflammation and oxidative stress in COVID-19 and AS comorbidity have not been investigated.

In addition to anti-inflammatory factors, other hub genes are involved in the progression of COVID-19 or AS. Researchers also used network pharmacology and molecular docking to identify AKT1 as a promising drug target for reducing tissue damage during COVID-19.^[66]^ As a biomarker, AKT1 was able to regulate pulmonary fibrosis through transforming growth factor-β pathways and participate in autophagy.^[67,68]^ In addition, *TP53* gene therapy could be used to treat COVID-19.^[69]^ TP53-induced glycolysis and apoptosis regulator is a fructose-2,6-bisphosphatase, which mitigates the development of AS by reducing the rate of glycolysis and protecting against oxidative stress.^[70]^ CASP3 belongs to the family of cysteine-aspartate proteases (or caspases).^[71]^ These caspases act as terminal executors of endogenous and exogenous apoptosis, and are involved in the progression of a variety of diseases. CAT is a major antioxidant enzyme in the vascular wall, which is also commonly used as a biomarker of free radical production in studies of AS.^[72]^ CAT can prevent oxidative damage cooperatively at different sites during ROS metabolic pathway activation.^[72]^ Moreover, CAT has been shown to play a protective role in pulmonary fibrosis by protecting lung epithelial cells from hydrogen peroxide–induced apoptosis, thereby reducing susceptibility to COVID-19.^[73,74]^ As a classical glycolytic enzyme, GAPDH is involved in cytosolic energy production. Moreover, GAPDH may have additional functions, which are independent of its well-documented role in glycolysis. GAPDH may therefore participate in the development of AS plaques.^[75]^ Ebrahimi et al^[76]^ proposed that inhibiting GAPDH in individuals with a weakened cellular innate immune response (e.g., in older people) may help treat viral diseases such as COVID-19. *INS* encodes insulin, a peptide hormone that plays a vital role in the regulation of carbohydrate and lipid metabolism. In patients with COVID-19/diabetes comorbidity, antidiabetic drugs and insulin therapy have to be adapted accordingly.^[77]^ In summary, the hub genes that we selected are closely related to a variety of key physiological and pathological processes, such as inflammation, oxidative stress, apoptosis, and immunity. To date, studies of the mechanism underlying the action of geniposide have mainly focused on its anti-inflammatory function; therefore further research is needed to explore the relationship between geniposide and other cellular processes.

### 4.3. Future prospects relating to the use of geniposide in the treatment of COVID-19 and AS comorbidity

Researchers have assessed that COVID-19 may be associated with subclinical acute and may be reversible AS recently,^[78]^ indicating the important of aggressive treatment. A Mendelian randomization showed that higher kidney injury molecule-1 reduced the likelihood of hospitalization by searching the association of cardiometabolic risk factors with COVID-19 severity.^[79]^ Inadequately, endogenous factors are more favorable as biomarkers than therapeutics. We have constructed an mRNA-miRNA network to identify changes happening at the transcriptional level and found that hsa-miR-34a-5p was most closely associated with the hub genes. Based on functional enrichment analysis, miR-34a-5p was identified as one of regulators of mRNA targets involved in the endothelial and inflammatory signaling pathways, as well as in viral diseases.^[80]^ miR-34a-5p was detected at lower expression levels in lung biopsies of COVID-19 patients, where it was involved in lung injury and immunothrombosis.^[81]^ Analysis of bronchial aspirate from critically ill patients with COVID-19 implied that COVID-19 induced a specific miRNAs signature, which included miR-34a-5p.^[82]^ Multi-level systems biology analysis has confirmed that hsa-miR-34a-5p is a predictive biomarker of COVID-19.^[83,84]^ In addition, the expression of miR-34a-5p during incident ischemic stroke may be a possible risk factor for secondary cardiovascular events.^[85]^ The key function of circulatory miR-34a-5p in endothelial dysfunction-linked cardiovascular pathology also offers novel routes for diagnosis.^[86]^ The expression level of miR-34a was lower in the tissues of patients with coronary artery disease than in normal arterial tissues, suggesting that miR-34a might promote vascular smooth muscle cell growth and migration.^[87]^ In the present study, screened miRNA and target gene interactions formed a putative regulatory network; validation of these predicted interactions should be performed in future studies. In addition, no studies have evaluated the action of geniposide at the molecular level.

Although our study provides new insights into the application of miRNAs and hub genes as biomarkers in COVID-19 and AS comorbidity, it had several limitations. For instance, it remains uncertain whether alterations in the levels of proposed biomarkers could be predictive of or responsive to disease development. The majority of miRNA and hub gene interactions identified in mRNA-miRNA regulatory network were putative and require in vitro and in vivo validation. Moreover, differences in the sex, age, habits, and medical history between patients and healthy controls could influence the response to geniposide treatment. Therefore, the use of clinical trials should also be considered in future studies.

## 5. Conclusions

In this study, systematic pharmacology and bioinformatics were combined to explore the possible mechanism underlying the use of geniposide in the treatment of patients with COVID-19/AS. Our results suggest that geniposide may regulate oxidative stress in patients with COVID-19/AS by modulating the FoxO signaling pathway. The results of this study provide a preliminary research direction for identifying the potential targets of geniposide in the treatment of COVID-19 and AS comorbidity; however, further experimental validation is needed. In the next study, we will use multi-omics to characterize a co-culture of CoV-infected macrophages and ox-low density lipoprotein-induced foam cells, simulating COVID-19/AS in vitro, to further explore the therapeutic mechanism of geniposide in these diseases.

## Acknowledgments

The authors thank the GEO datasets for providing the data.

## Author contributions

**Formal analysis:** Lijin Qing.

**Investigation:** Lijin Qing.

**Methodology:** Lijin Qing.

**Software:** Lijin Qing.

**Writing – original draft:** Lijin Qing.

**Writing – review & editing:** Lijin Qing.

**Conceptualization:** Wei Wu.

**Funding acquisition:** Wei Wu.

**Project administration:** Wei Wu.

## Supplementary Material




