# Supplementary material for: The mechanism of geniposide in patients with COVID-19 and atherosclerosis: A pharmacological and bioinformatics analysis
Source: Medicine (Baltimore). 2024 Aug 2;103(31):e39065. doi: 10.1097/MD.0000000000039065 (PMC11296471; doi:10.1097/MD.0000000000039065)
Supplement: Supplementary file 1 [file medi-103-e39065-s001.docx]

| Gene ID |  |  |  |  |  |  |
| --- | --- | --- | --- | --- | --- | --- |
| MMP7 | UBASH3B | EPHX2 | MAPKAPK2 | MCL1 | NOS2 | RAB27B |
| TF | IGHG1 | FAP | BCL2 | TLR4 | CCNE1 | SPEN |
| EGFR | MKI67 | TRIM21 | STAT5A | RARB | ALAD | VAV2 |
| SLC6A2 | CA13 | HK2 | MMP1 | CA5A | CA9 | HSP90AA1 |
| CA14 | NUP214 | LGALS9 | CA12 | GAST | GCGR | SLC28A2 |
| CPSF3 | ADORA2A | CA2 | MMP3 | GPT | FLI1 | MAG |
| HK1 | MYBL2 | GSK3A | ZPR1 | INS | GSK3B | ETS1 |
| CA7 | TBX1 | TOP1 | PEBP1 | GSTM1 | TRIP10 | NR1H4 |
| PYGL | MPO | CFTR | GLP1R | IGKC | ATIC | SATB2 |
| IL1B | DIO2 | PHGDH | LGALS2 | MMP14 | UEVLD | ARSB |
| RNF8 | PATJ | MMP8 | IGFBP3 | TYR | BAX | KAT2A |
| VWF | MGMT | OCLN | LSS | PNP | P2RX3 | CASP3 |
| RAN | IL6 | SOD1 | TRAF4 | CLK1 | MAD2L1 | NGLY1 |
| EEF1A1 | PMP2 | METAP2 | TPI1 | TTR | MME | OGA |
| SLC5A1 | TOP2A | SI | EDNRA | CDK5R1 | MAGI1 | PTGES |
| MMP9 | HPRT1 | TRIO | TNF | LDHB | ESR1 | PABPC1 |
| EIF5A | STRBP | MAPK9 | SST | MLN | GAP43 | DAO |
| AMPD3 | INSR | TCF7L2 | DAG1 | SEC13 | MMP12 | HSPA8 |
| IL10 | AKR1C3 | KPNB1 | CAT | PDK4 | GGH | CHN2 |
| VPS4B | IMPDH2 | TKT | CNDP1 | HMOX1 | LGALS7 | IL4I1 |
| LGALS3 | PRDM16 | CTNNB1 | IMPDH1 | GBP1 | AKR1B1 | CRMP1 |
| TAGLN3 | SENP7 | SNX9 | PFKFB4 | SLC29A1 | TST | LYZ |
| ADORA2B | GAA | PDX1 | HNRNPA1 | TP53 | REPS1 | ADA |
| DEK | TGFB1 | CA3 | SLC30A9 | GTF2I | EPAS1 | BCL2L1 |
| TYMP | BCL2A1 | ADAM17 | EPHA2 | DOK2 | GSTM2 | MAPK3 |
| ACTR3 | CA4 | GAPDH | FOLH1 | EIF4H | RPA1 | ITPKC |
| CHEK1 | FBP1 | ALK | GPI | IDE | UAP1 | RABGGTA |
| SELE | FUCA1 | MTOR | LCK | ANXA1 | MMP2 | GRK1 |
| ANXA6 | SLC28A3 | FLNB | CYP2E1 | PTPN11 | UQCRC1 | TNFRSF9 |
| CIDEA | ZBTB43 | SEC23A | PPP2R1A | DPP4 | PPARGC1A | MAPK10 |
| MGAM | GBA | HSPA5 | COL2A1 | QARS1 | ANXA8 | UQCRQ |
| HRAS | FOXO1 | ALOX12 | ACR | CD28 | ABL1 | MMP13 |
| ESD | TK1 | BAP1 | VCAM1 | PDCD11 | GCG | VAV1 |
| ELOVL3 | AKT1 | PYGM | CA6 | AR | TLN1 | SORT1 |
| ADK | ZEB2 | CA1 | COG2 | RAB31 | ADORA1 | SLC5A2 |
| PFKFB3 | ANXA4 |  |  |  |  |  |

Supplementary Table S1 : Shared target genes between the geniposide, COVID-19, and AS.
