# Supplementary material for: The mechanism of geniposide in patients with COVID-19 and atherosclerosis: A pharmacological and bioinformatics analysis
Source: Medicine (Baltimore). 2024 Aug 2;103(31):e39065. doi: 10.1097/MD.0000000000039065 (PMC11296471; doi:10.1097/MD.0000000000039065)
Supplement: Supplementary file 2 [file medi-103-e39065-s002.docx]

| Terms | Adjusted P-value | | Genes |
| --- | --- | --- | --- |
| hsa-miR-34a-5p | 0.004243648 | CASP3;AKT1;TNF;TP53;MAPK3 | |
| hsa-miR-451a | 0.015417238 | IL6;AKT1 | |
| hsa-miR-26a-1-3p | 0.039287818 | CASP3;TP53 | |
| hsa-miR-155-5p | 0.039287818 | IL6;CASP3;CAT;AKT1 | |
| hsa-miR-30b-5p | 0.039287818 | CASP3;CAT;TP53 | |
| hsa-miR-223-3p | 0.039287818 | IL6;TP53 | |
| hsa-miR-125b-5p | 0.039287818 | AKT1;TNF;TP53 | |
| hsa-miR-375 | 0.045562689 | CASP3;TP53;MAPK3 | |
| hsa-miR-365a-3p | 0.045562689 | IL6;AKT1 | |
| hsa-miR-138-5p | 0.045562689 | CASP3;AKT1 | |

Supplementary Table S2 : Results of mRNA-miRNA network.
